# A study on the prevention of hemorrhage and perforation in patients with primary gastric diffuse large‐B cell lymphoma during treatment with immunochemotherapy

**DOI:** 10.1002/cam4.5486

**Published:** 2023-01-09

**Authors:** Limei Zhang, He Huang, Zhao Wang, Xiaojie Fang, Huangming Hong, Yungchang Chen, Quanguang Ren, Yuyi Yao, Zegeng Chen, Fei Pan, Xiaoqian Li, Meiting Chen, Tongyu Lin

**Affiliations:** ^1^ Department of Medical Oncology Sun Yat‐sen University Cancer Center, State Key Laboratory of Oncology in Southern China, and Collaborative Innovation Center of Cancer Medicine Guangzhou China; ^2^ Department of Medical Oncology Sichuan Cancer Hospital and Institute, Sichuan Cancer Center, School of Medicine, University of Electronic Science and Technology of China Chengdu China; ^3^ Department of Oncology Tongji Hospital, Tongji Medical College, Huazhong University of Science and Technology Wuhan China

**Keywords:** hemorrhage, perforation, primary gastric diffuse large B‐cell lymphoma, R‐CHOP

## Abstract

**Background:**

Stomach hemorrhage and perforation are very severe and common complications in patients with primary gastric diffuse large B‐cell lymphoma (PG‐DLBCL) during treatment with immunochemotherapy. However, no relevant clinical studies have been performed on the prevention of these serious complications.

**Methods:**

Patients diagnosed with PG‐DLBCL were enrolled in this retrospective study. The prevention group received standard rituximab, cyclophosphamide, doxorubicin, vincristine and prednisone (R‐CHOP) treatment without prednisone combined with antacids and anti‐Helicobacter pylori (Hp) therapy. These patients received R‐CHOP‐based treatment until the complete recovery of gastric ulcers, as proven by gastroscopy. The control group received a standard R‐CHOP regimen. Toxicity and survival were the main endpoints.

**Results:**

A total of 52 patients received preventative treatment, while 146 patients did not. Among patients with stage I, II‐1, and II‐2 disease, the prevention group had a lower rate of hemorrhage and perforation (0/40) than the control group (10/78, *p* = 0.044). At a median follow‐up time of 25 months, the 5‐year event‐free survival (EFS) rates were 97.1% in the prevention group and 66.1% in the control group (*p* = 0.025), and the 5‐year overall survival (OS) rates were 100% and 72.0%, respectively (*p* = 0.021). However, the differences in the 5‐year EFS and OS of patients with disseminated disease were not statistically significant.

**Conclusions:**

Preventative treatment can decrease the risk of hemorrhage and perforation in patients with localized PG‐DLBCL during immunochemotherapy, leading to better EFS and OS in these patients. However, preventative treatment failed to reduce the risk of gastric hemorrhage and perforation and did not improve survival (EFS and OS) in advanced‐stage patients.

## Graphical abstract

Preventative R‐CHOP (rituximab, cyclophosphamide, doxorubicin, vincristine and prednisone) can decrease the risk of hemorrhage and perforation in patients with localized primary gastric diffuse large‐B cell lymphoma during immunochemotherapy, leading to better survival in these patients. However, preventative treatment failed to achieve similar effect in advanced‐stage patients.

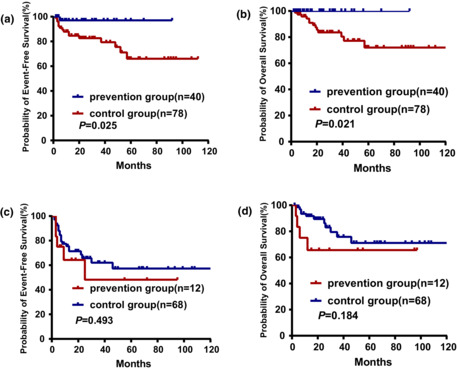

## 1 INTRODUCTION

Primary gastric diffuse large B‐cell lymphoma (PG‐DLBCL) accounts for 1% ~ 7% of gastric malignancies^1^ and for approximately 50% of gastric lymphoma cases.^2^, ^3^ To date, there is still no standard treatment for PG‐DLBCL. Currently, rituximab, cyclophosphamide, doxorubicin, vincristine and prednisone (R‐CHOP) followed by involved‐field radiotherapy (IFRT) is considered a standard regimen for treating localized PG‐DLBCL,^4^, ^5^ although the data supporting this regimen were extrapolated from the results of patients with nodal DLBCL.^6^ For advanced or disseminated disease, six to eight cycles of R‐CHOP are usually recommended. Treatment with hemotherapy alone or sequential treatment with chemotherapy and radiotherapy can achieve the same therapeutic effect, and surgical resection has little benefit in the treatment of primary gastric lymphoma.^7^

Hemorrhage and perforation are serious complications of gastric lymphoma and are more highly associated with aggressive lymphomas^8^; these complications can appear as the initial presentation of gastric lymphoma or after chemotherapy. Surgery is not a recommendation for patients with treatment‐naïve unselected gastric lymphoma, but tumor reduction surgery may be considered to decrease the risk of gastric bleeding and perforation associated with chemotherapy. In addition, proton pump inhibitors (PPIs) can increase the pH of the gastric juice, thereby stabilizing blood clots and improving clinical outcomes. The use of a PPI decreases the risk of rebleeding and is required for patients undergoing surgery after ulcer bleeding.^9^ Helicobacter pylori (Hp) infection increases the risk of peptic ulcer bleeding.^10^ Gastric lymphoma often develops from ulcers in the stomach. Therefore, the eradication of H.p can decrease the risk of bleeding and perforation in patients with gastric lymphoma. Corticosteroids such as prednisone increase the risk of peptic ulcers and gastrointestinal hemorrhage,^11^ especially when a high dose of prednisone is used before an ulcer is cured.

Therefore, we retrospectively collected and analyzed data from patients who were newly diagnosed with PG‐DLBCL to explore effective measures for decreasing the risk of hemorrhage and perforation in these patients and to improve survival.

## 2 PATIENTS AND METHODS

### 2.1 Patients

Patients who were diagnosed with gastric DLBCL and who were undergoing treatment with the R‐CHOP regimen between 2002 and 2018 at Sun Yat‐sen University Cancer Center were analyzed. The WHO classification criteria were used to histologically identify PG‐DLBCL. The best method for distinguishing PG‐DLBCL from systemic DLBCL involving the stomach is unclear. Patients with predominant gastric lesions were considered to have PG‐DLBCL according to a definition provided by previous studies.^12^, ^13^ All patients were older than 18 years old, and patients who underwent surgery due to gastric lymphoma before being treated with chemotherapy were not included in this study.

Patients who received standard R‐CHOP treatment without prednisone combined with antacids and anti‐Hp therapy were allocated to the prevention group, and these patients received R‐CHOP‐based treatment until the complete recovery of gastric ulcers was proven by gastroscopy. The time of the start of prednisone was different for every patient in the prevention group, which mainly depended on the time of the complete recovery of gastric ulcers proven by gastroscopy. After every cycle of chemotherapy, these patients underwent gastroscopy examination to check whether the gastric ulcers had recovered. If the gastric ulcers obtained complete recovery, the patients could receive a standard R‐CHOP regimen. Therefore, some patients started to use prednisone in the second, third or fourth cycle of R‐CHOP. Regarding the use of PPIs, when patients received R‐CHOP chemotherapy in the hospital, they received intravenous proton pump inhibitors (e.g., omeprazole 40 mg iv qd). In addition, the anti‐Hp drugs were only given to patients who had Hp infection in the prevention group. When the patients were diagnosed with Hp infection, they received anti‐Hp therapy as soon as possible. The anti‐Hp therapy contained oral proton pump inhibitors (PPIs), bismuth, two kinds of antibiotics (metronidazole and tetracycline or clarithromycin and amoxicillin) (quadruple therapy), which lasted approximately 2 weeks. During chemotherapy, if patients do not finish anti‐Hp therapy, they should still continue the anti‐Hp therapy. Patients who failed eradication received a second‐line antibiotic therapy following local guidelines.

The control group received a standard R‐CHOP regimen. For anti‐allergy reactions, anti‐allergy medications are usually adopted before rituximab transfusion in both groups, including glucocorticoids (e.g., dexamethasone), diphenhydramine, antipyretic drugs and analgesics. For the use of doxorubicin, 50 mg/m^2^ on day 1, every 21 days. If patients received surgery because of gastric bleeding or perforation, after surgery, these patients still continued to receive chemotherapy if they did not finish chemotherapy. When patients completely recovered after surgery and were in good condition, they continued to receive chemotherapy at a standard dose without any reduction. Prednisone is not avoided in the following treatment. We defined a gastric bleeding event as patients developing melena, hematemesis, or a sudden decrease in hemoglobin level (>2 g/dL in 1 week) with endoscopic findings of stigmata of active or recent hemorrhage. The response to treatment was evaluated based on the WHO criteria. Not all patients underwent endoscopic ultrasonography (EUS) at baseline for the staging evaluation. The Lugano staging system was used for patient staging,^14^ and the tumors were classified as follows: stage I, tumor limited to the stomach; stage II, tumor extending to local (II‐1) or distant (II‐2) nodes; stage II‐E, tumor involving adjacent organs or tissues; and stage IV, disseminated extranodal involvement or concomitant supradiaphragmatic lymph node involvement. All the data were obtained from the medical records at this center.

### 2.2 Statistical analysis

The Kaplan–Meier method was used to estimate survival. We used the log‐rank test for comparisons between two groups. The χ^2^ test was used to evaluate the relationships between the clinical characteristics and results in the two groups. We considered a two‐sided *p* value <0.05 to be significant. All statistical analyses were performed with the SPSS software package, version 22 and GraphPad Prism 7. Event‐free survival (EFS) was measured from the date of chemotherapy initiation to the date of primary treatment failure, severe complications (hemorrhage events and perforations), first disease progression, relapse, or death due to any cause. Overall survival (OS) was defined as the time from the date of chemotherapy initiation to the date of the final follow‐up or death due to any cause.

## 3 RESULTS

### 3.1 Patient characteristics

**FIGURE 1:**
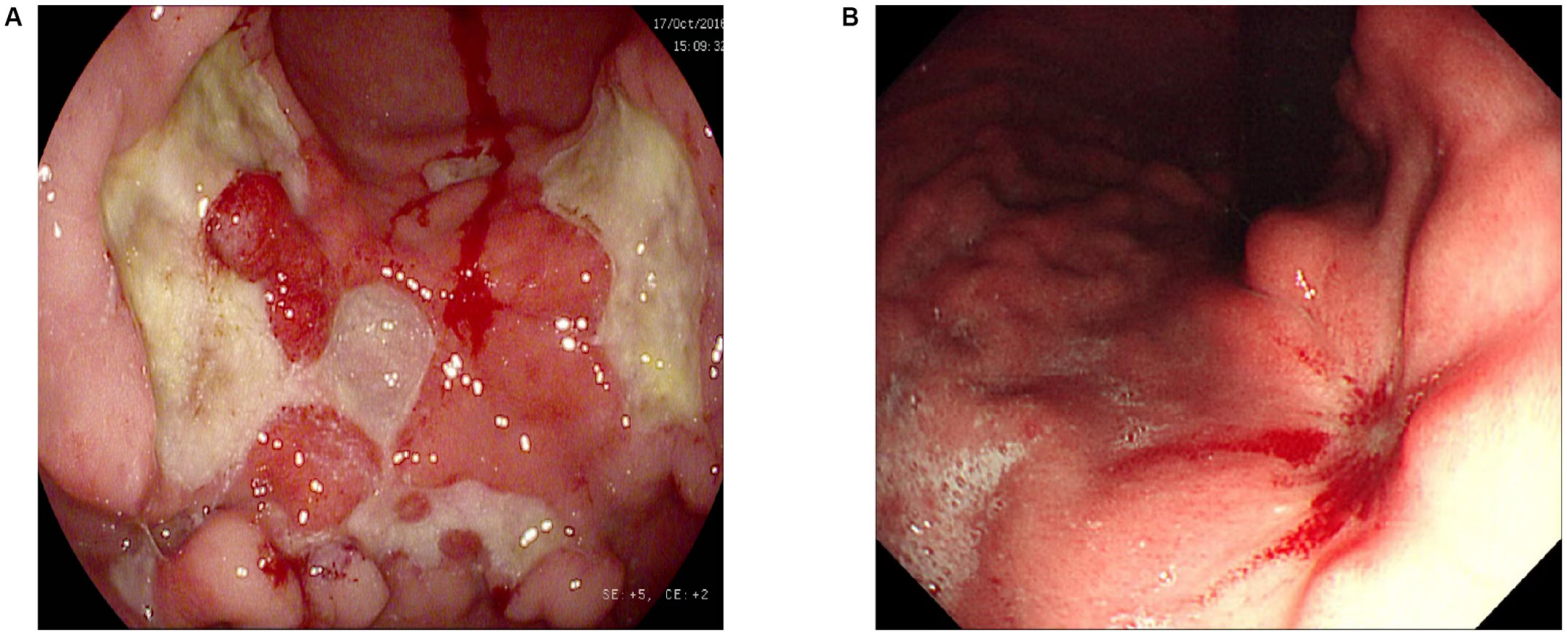
Endoscopic images of gastric diffuse large B cell lymphoma. (A) Lymphoma at the greater curvature of the stomach, and the surrounding mucosa presented embankment like uplift, accompanied by bleeding; (B) The lymphoma in the lesser curvature of the body of the stomach was bleeding after chemotherapy.

The baseline characteristics of the 198 patients who were included in this study are shown in Table 1. Between 2002 and 2018, 52 patients received preventative treatment, while 146 patients did not. The median follow‐up time of this study was 25 months (range 2–112 months). The median age of the patients was 53 years (range 21–86 years), and the female‐to‐male ratio was 1:0.96. The majority of patients had a good performance status (≤Eastern Cooperative Oncology Group (ECOG) grade 0/1, 98%), and 56.6% of the patients were infected with HP. The clinical characteristics of the two groups were compared, including sex, age, B symptoms, lactate dehydrogenase (LDH), ECOG performance status, Lugano stage, International Prognostic Index (IPI), pathological characteristics, HP infection rate and extranodal involvement. The clinical characteristics between the two groups were balanced and comparable. Gastroscopy images of primary gastric diffuse large B‐cell lymphoma are shown in Figure 1.

**TABLE 1:** Clinical characteristics of all patients (*N* = 198)

Characteristic	All	Prevention group	Control group	*p* Value
	*N* = 198	*N* = 52	*N* = 146	
		No.(%)	No.(%)	
Median	53(21‐86)	53.00(25‐73)	52.50(21‐86)	0.518
Age(range), years				
≤60	137(69.2)	37(71.2)	100(68.5)	0.721
>60	61(30.8)	15(28.8)	46(31.5)	
Sex				
Male	97(49.0)	24(46.2)	73(50.0)	0.634
Female	101(51.0)	28(53.8)	73(50.0)	
Performance status				
ECOG 0/1	194(98.0)	52(100.0)	142(97.3)	0.527
ECOG≥2	4(2.0)	0	4(2.7)	
Serum LDH level				
Normal	134(67.7)	37(71.2)	97(66.4)	0.704
Increased	60(30.3)	15(28.8)	45(30.8)	
Missing	4.(2.5)	0	4(2.7)	
B symptoms				
Absent	148(74.7)	44(84.6)	104(71.2)	0.065
Present	49(24.7)	8(15.4)	41(28.1)	
Missing	1(0.5)	0	1(0.7)	
IPI				
0–1	139(70.2)	37(71.2)	102(69.9)	0.926
≥2	55(27.8)	15(28.8)	40(27.4)	
Missing	4(2.0)	0	4(2.7)	
Lugano stage				
I–II	140(70.6)	40(76.9)	100(68.5)	0.251
IV	58(29.4)	12(23.1)	46(31.5)	
Perforation or hemorrhage				
Absent	181(91.4)	52(100)	129(88.4)	**0.022**
Present	17(8.6)	0	17(11.6)	
Hp infection				
(+)	112(56.6)	37(71.2)	75(51.4)	0.641
(−)	38(19.2)	11(21.2)	27(18.5)	
Missing	48(24.2)	4(7.6)	44(30.1)	
Hans's subtype				
GCB	69(34.8)	18(34.6)	51(34.9)	0.240
nGCB	83(41.9)	29(55.8)	54(37.0)	
Missing	46(23.2)	5(9.6)	41(28.1)	
Extranodal involvement				
<2	185(93.4)	49(94.2)	136(93.2)	1.000
≥2	13(6.6)	3(5.8)	10(6.8)	

Abbreviations: ECOG, Eastern Cooperative Oncology Group; GCB, germinal center B‐cell; Hp, Helicobacter pylori; IPI, international prognostic index; LDH, lactate dehydrogenase.

Bold indicates significance level at p‐value *p* value <0.05

### 3.2 Treatment outcomes of all patients

**FIGURE 2:**
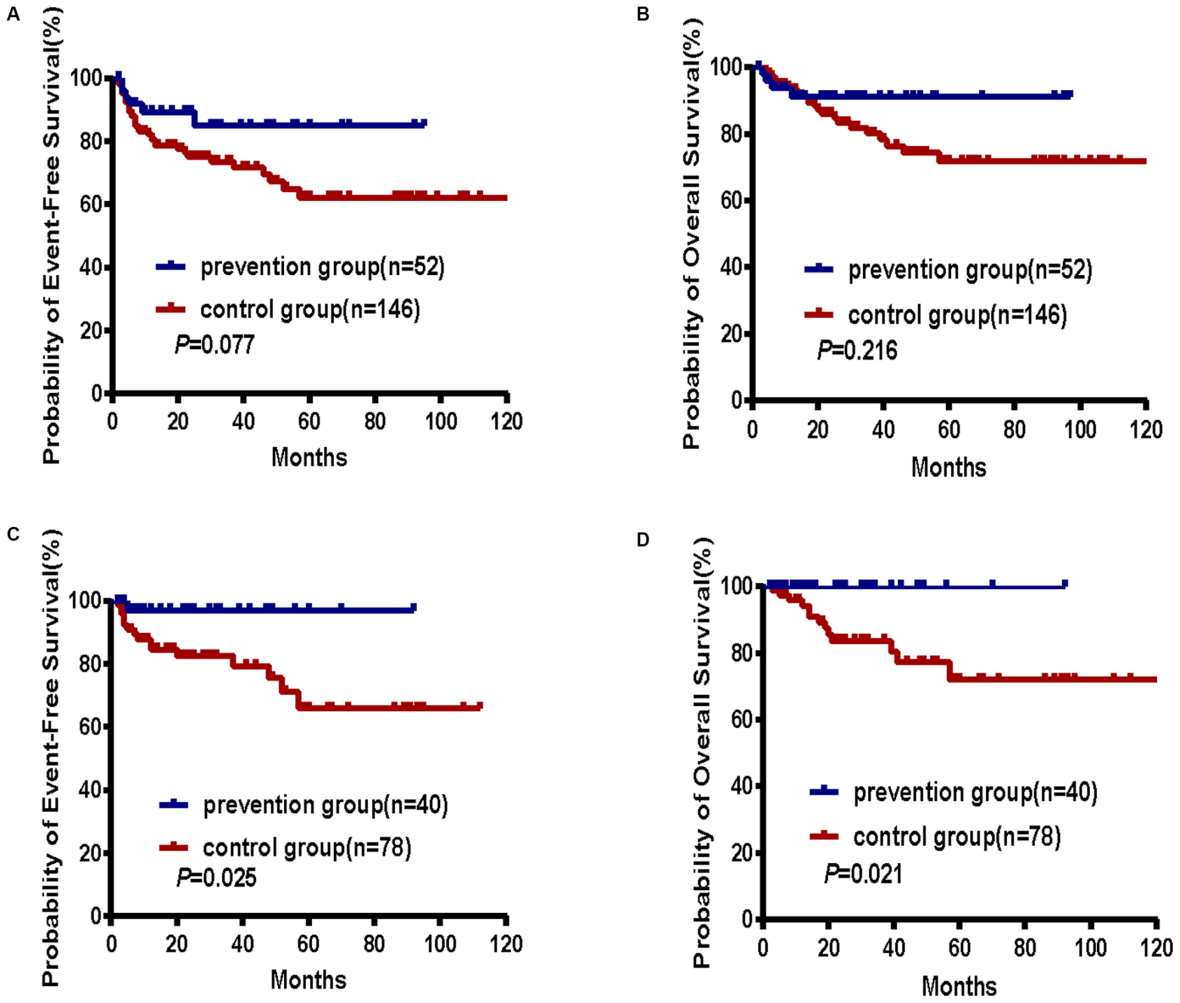
Survival outcomes were compared in patients according to the treatment strategy. EFS (A) and OS (B) in all patients(*N* = 198), EFS (C) and OS (D) in stage I, II‐1, and II‐2 patients (*N* = 118).

Among the 198 patients, the rate of hemorrhage and perforation in the prevention group (0/52) was lower than that in the control group (17/146, *p* = 0.022). Four hemorrhages and 13 perforations occurred in the control group. Perforation simultaneously occurred in both the small intestine and stomach in one patient. Thirty‐seven 5‐year EFS events occurred in the control group, while 6 events occurred in the prevention group. Thus, the 5‐year EFS rates were 62.2% in the control group and 85.0% in the prevention group (*p* = 0.077, Figure 2A). For 5‐year OS, 24 events occurred in the control group, while 4 events occurred in the prevention group. Thus, the 5‐year OS rates were 71.7% and 91.1%, respectively (*p* = 0.216, Figure 2B).

### 3.3 Treatment outcomes of patients with stage I, II‐1, and II‐2 disease

The characteristics of the 118 patients with localized disease are shown in Table 2. The clinical characteristics between the two groups were almost balanced and comparable, except for B symptoms. Forty patients (Lugano stage I, II‐1, and II‐2) received preventative treatment, while 78 patients with the same disease stages did not. A complete or unconfirmed complete response was observed in 92% (35/38) of the evaluable patients in the prevention group and in 85% (63/74) of the evaluable patients in the control group. Sixteen 5‐year EFS events (primary treatment failure, severe complications, progression, relapse, or death) were observed in the control group, and 1 5‐year EFS event was observed in the prevention group (in 20.5% and 2.5% of patients, respectively). The EFS was significantly longer for patients receiving preventative treatment than for patients in the control group. The 5‐year EFS rates were 66.1% in the control group and 97.1% in the prevention group (*p* = 0.025, Figure 2C). Regarding OS, 13 events occurred in the control group, while no events occurred in the prevention group. Thus, the 5‐year OS rates were 72.0% and 100%, respectively (*p* = 0.021, Figure 2D).

**TABLE 2:** Comparison of patients based on treatment strategy (*N* = 118)

Characteristic	Prevention group	Control group	*p* Value
	*N* = 40	*N* = 78	
	No.(%)	No.(%)	
Median age (range), years	51.78	51.27	0.843
≤60	29(72.5)	54(69.2)	0.713
>60	11(27.5)	24(30.8)	
Sex			
Male	18(45.0)	39(50.0)	0.607
Female	22(55.0)	39(50.0)	
Performance status			
ECOG 0/1	40(100.0)	77(98.7)	1.000
ECOG≥2	0(0)	1(1.3)	
Serum LDH level			
Normal	31(77.5)	58(74.4)	0.914
Increased	9(22.5)	19(24.4)	
Missing	0	1(1.3)	
B symptoms			
Absent	37(92.5)	58(74.4)	**0.024**
Present	3(7.5)	19(24.4)	
Missing	0(0)	1(1.3)	
IPI			
0–1	35(87.5)	70(89.7)	0.478
≥2	5(12.5)	5(6.4)	
Missing	0	3(3.8)	
Lugano stage			
I	24(60.0)	51(65.4)	0.565
II‐1/II‐2	16(40.0)	27(34.6)	
Perforation or hemorrhage			
Absent	40(100.0)	68(87.2)	**0.044**
Present	0(0)	10(12.8)	
Hp infection			
(+)	31(77.5)	42(53.8)	0.513
(−)	8(20.0)	15(19.2)	
Missing	1(2.5)	21(26.9)	
Hans's subtype			
GCB	15(37.5)	33(42.3)	0.100
nGCB	22(55.0)	24(30.8)	
Missing	3(7.5)	21(26.9)	

### 3.4 Treatment outcomes of patients with stage II‐E and IV disease

**FIGURE 3:**
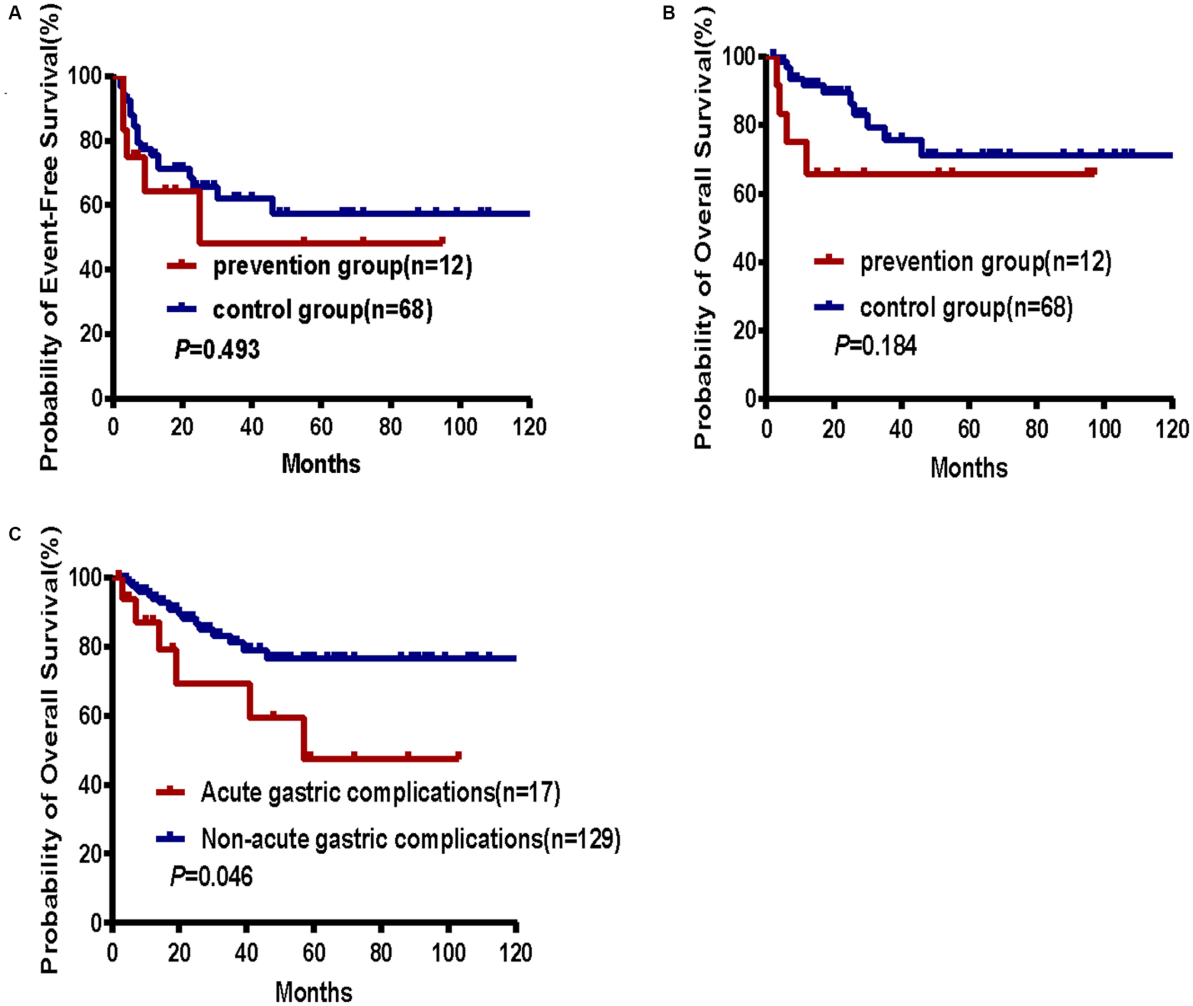
EFS (A) and OS (B) in stage II‐E and IV patients(*N* = 80). OS (C) in patients with acute gastric complications in the control group (*N* = 146).

Twelve patients with stage II‐E and IV disease received preventative treatment, while 68 patients with the same disease stages did not receive preventative treatment. A complete or unconfirmed complete response (CR) was observed in 70% (7/10) of the evaluable patients in the prevention group and in 69.2% (45/65) of the evaluable patients in the control group. Twenty‐one 5‐year EFS events (primary treatment failure, severe complications, progression, relapse, or death) were observed in the control group, and 5 5‐year EFS events were observed in the prevention group (in 30.9% and 41.7% of patients, respectively). The 5‐year EFS rates were 57.3% in the control group and 48.2% in the prevention group (*p* = 0.493, Figure 3A). For OS, 11 events occurred in the control group, while 4 events occurred in the prevention group. Thus, the 5‐year OS rates were 71.1% and 65.6%, respectively (*p* = 0.184, Figure 3B). The differences between the 5‐year EFS and OS were not statistically significant.

### 3.5 Complications of localized disease (stage I, II‐1, and II‐2)

The rates of complications, including hemorrhage and perforation, were quite different between the two groups. The rate of gastric hemorrhage and perforation was significantly higher in the control group (patients who received the standard CHOP regimen) than in the prevention group (12.8% vs. 0, *p* = 0.044). Of these patients, 12.8% (10 of 78) developed gastric hemorrhage and perforation, and 100% (10 of 78) of these events occurred after chemotherapy. The median day of hemorrhage and perforation after the initiation of chemotherapy was 25 days (mean, 53 days; range, 4–221). Of these patients, 40% (4 of 10) experienced gastric hemorrhage alone, and 60% (6 of 10) experienced gastric perforation alone. Among these patients, four patients chose conservative treatment (3 patients with perforations and 1 patient with hemorrhage). Of the four patients who chose conservative treatment, one patient had surgical indications, but she stopped follow‐up treatment because of financial problems, and she eventually died. One patient received total gastrectomy because of gastric bleeding, and one patient initially received conservative treatment but then underwent palliative resection of the gastric fundus and cardiac masses because of gastric perforation. The other four patients received subtotal gastrectomy (2 patients with perforations and 2 patients with hemorrhage). Five of these 10 patients eventually died. The characteristics of these patients who developed gastric perforation or hemorrhage during chemotherapy are shown in Table 3.

**TABLE 3:** Characteristics of patients with stage I/II‐1/II‐2 disease developed gastric perforation or hemorrhage during chemotherapy

No	AGC	treatment	Status
1	hemorrhage	Conservative treatment	Alive
2	perforation	Conservative treatment	Alive
3	perforation	Conservative treatment	Dead
4	perforation	Conservative treatment	Alive
5	hemorrhage	total gastrectomy	Dead
6	hemorrhage	Subtotal gastrectomy	Alive
7	hemorrhage	Subtotal gastrectomy	Alive
8	perforation	Palliative gastric fundus and cardia lumpectomy	Dead
9	perforation	Subtotal gastrectomy	Dead
10	perforation	Subtotal gastrectomy	Dead

Abbreviation: AGC, actue gatric complications.

### 3.6 Toxicity

Table 4 shows the main adverse events that occurred in each group. The most common toxicities were granulocytopenia of all grades (6.8% in the control group and 7.7% in the prevention group, *p* = 0.0839), thrombocytopenia of all grades (8.2% in the control group and 9.6% in the prevention group, *p* = 0.984) and nausea/vomiting of all grades (8.9% in the control group and 7.7% in the prevention group, *p* = 0.789) (Table 4). Toxicities occurred with a similar frequency in both groups. No obvious cardiac, hepatic or renal toxicity was observed in either group. In addition, the overall survival of patients who experienced acute gastric complications (hemorrhage and perforation) was worse than that of those without acute gastric complications (*p* = 0.046, Figure 3C).

**TABLE 4:** Main adverse events in each group (*N* = 198)

	Any grade			Grade3/4		
	Control group n(%)	Prevention group n(%)	*p* Value	Control group n(%)	Prevention group n(%)	*p* Value
Hematological						
Hemoglobin level	5(3.4)	2(3.8)	0.888	3(2.1)	0	0.704
Neutrophil count	10(6.8)	4(7.7)	0.839	8(5.5)	3(5.8)	0.938
Platelet count	12(8.2)	5(9.6)	0.984	8(5.5)	3(5.8)	0.938
Gastrointestinal						
Nausea/vomiting	13(8.9)	4(7.7)	0.789	1(0.7)	1(1.9)	0.443
Diarrhea	5(3.4)	2(3.8)	0.888	0	0	–
Stomatitis	4(2.7)	2(3.8)	0.689	1(0.7)	0	0.550
Dysphagia	2(1.4)	0	0.967	0	0	–
Other						
Fever	18(12.3)	7(13.5)	0.833	0	0	–
Allergic reaction	1(0.7)	0	0.550	0	0	–
Paresthesia	9(6.2)	3(5.8)	0.918	0	0	–
Cough	3(2.1)	0	0.704	0	0	–

## 4 DISCUSSION

According to a previous study, the incidence of gastric perforation in gastric DLBCL patients was 6%, which included patients with different disease stages. However, due to different chemotherapy regimens and small sample sizes, some hospitals observed higher rates of gastric perforation or bleeding. In our study, among patients with stage I, II‐1, and II‐2 disease, the rate of gastric hemorrhage and perforation was significantly higher in the control group than in the prevention group (12.8% vs. 0, *p* = 0.044). Our results suggest that preventive treatment can reduce the risk of gastric hemorrhage and perforation in these patients. Among patients with stage I, II‐1, and IL‐2 disease, the complete response rate (CR) in the prevention group was 92.1% higher than that in the control group (85.0%, *p* = 0.451), indicating that preventive treatment did not lead to disease progression in early stages. Early ulcer healing may also allow patients to have better nutrition and immunity. As a result, early ulcer healing can improve patient tolerance for subsequent treatment. In these patients, the 5‐year EFS rates were 97.1% in the prevention group and 66.1% in the control group (*p* = 0.025). The 5‐year OS rates were 100% and 72%, respectively (*p* = 0.021). Our results indicate that decreasing the risk of hemorrhage and perforation by using preventative treatment during immunochemotherapy achieves a better EFS and OS in patients with localized PG‐DLBCL.

In our study, the patients in the prevention group received standard R‐CHOP treatment without prednisone combined with antacids and anti‐HP therapy, and these patients received R‐CHOP‐based treatment until the complete recovery of gastric ulcers was proven by gastroscopy. Glucocorticoids increase the risk for adverse gastrointestinal effects, including gastritis, ulcer formation, and gastrointestinal bleeding. The estimated relative risks associated with the use of glucocorticoids alone to treat gastrointestinal adverse effects vary from 1.1 (not significant) to 1.5 (marginally significant).^11^, ^15^ In addition to upper gastrointestinal morbidity, visceral perforation is another complication associated with glucocorticoid use.^7^, ^16^, ^17^ To our knowledge, the R‐CHOP regimen contains a high dose of prednisone, so the patients in the prevention group received standard R‐CHOP treatment without prednisone until the complete recovery of gastric ulcers. For antacids, PPI use results in faster control of peptic ulcers and higher ulcer healing rates.^18^, ^19^, ^20^ In addition, Wohrer et al reported that the use of PPIs in patients with gastric lymphoma can reduce the risk of chemotherapy‐related acute complications.^21^ In addition, PPIs are also included in antibiotic regimens for the treatment of Hp, which is the most common chronic bacterial infection, is related to many diseases, such as peptic ulcer disease, gastric adenocarcinoma, and gastric mucosa‐associated lymphoid tissue lymphoma (MALT).^22^, ^23^, ^24^, ^25^ In addition, the article by Ferreri et al, which described a phase 2 trial, showed that the eradication of H.p resulted in an excellent response rate in gastric DLBCL patients.^26^ In our study, for patients who were infected with H.p., anti‐H.p therapy promoted gastric ulcer healing.

For patients in the control group, once hemorrhage and perforation occurred, metastasis was accelerated, the next cycle of chemotherapy was delayed, the tolerance of patients for treatment was weakened, and the hospital stay was prolonged. In our opinion, preventative treatment decreased the risk of stomach hemorrhage and perforation, thus improving the survival of patients with stage I, II‐1, and II‐2 disease. However, when preventative treatment was used in patients with advanced disease (stage IIE/IV), the different treatment strategies did not result in significantly different OS and EFS rates between the two groups (*p* > 0.05). Preventative treatment did not significantly improve the survival of patients with advanced disease in this study. A possible limitation of the comparison of OS and EFS in patients with advanced disease (stage IIE/IV) in our study was that there were fewer patients in the prevention group than in the control group, which may have biased the results. In addition, for patients with advanced disease, preventative treatment was not as important because advanced‐stage disease had a greater influence on survival than the treatment strategy. Patients with disseminated disease are more likely to have refractory disease or to relapse, which leads to worse survival. For patients with advanced disease, receiving preventative treatment did not result in survival benefits, and a more effective regimen could be considered.

Currently, there is no standard regimen for the treatment of refractory/relapsed PG‐DLBCL, which usually refers to nodal DLBCL. Patients with refractory or relapsed lymphomas are recommended to receive systemic chemoimmunotherapy. Chemotherapy‐sensitive patients are recommended to undergo hematopoietic stem cell transplantation, while chemotherapy‐resistant patients are encouraged to participate in clinical trials or to receive allohematopoietic stem cell transplantation (allo‐HSCT) or chimeric antigen receptor T (CAR‐T) treatment. Promising results of CAR T‐cell therapies have been observed in patients with various B‐cell malignancies in many clinical trials. Therefore, CAR T‐cell therapies could be an option for treating refractory/relapsed PG‐DLBCL. Based on the results of the ZUMA‐1 study, CAR T‐cell therapy will be applied to the treatment of refractory DLBCL in the future.^27^, ^28^ In addition, targeted drugs such as venetoclax ibrutinib and idelalisib continue to be studied in patients with lymphomas, including PG‐DLBCL.

PPIs are effective drugs that inhibit acid secretion from the stomach. In addition, potassium‐competitive acid inhibitors (PACBs) are a new therapeutic strategy that inhibits the secretion of acid by the stomach and are available only in Asia.^29^, ^30^, ^31^, ^32^ Randomized controlled trials (RCTs) have shown that PACBs have efficacy and tolerability profiles that are similar to those of PPIs in promoting ulcer healing.^31^, ^32^, ^33^ Some studies have shown that PACBs plus antibiotics may be effective in the eradication of Hp.^34^, ^35^ Therefore, to reduce the risk of gastric hemorrhage and perforation, the addition of PACBs may be considered in the future.

Despite the innovation of this study mentioned above, there are still some limitations in our study. First, this study was conducted at a single center without a large number of patients, and it was a retrospective study, not a prospective and randomized study. Therefore, bias was inevitable. Second, the number of patients in the two groups was not similar. Third, endosonography can help not only in the diagnosis of the tumor, confirming the depth of tumor infiltration but also in the involvement of the perigastric lymph nodes. Several studies have proven that endosonography is highly accurate in early staging and assessing response to treatment as well as during follow‐up after treatment.^36^, ^37^ However, most patients with PG‐DLBCL did not undergo endosonography in this study. Therefore, multicenter, large‐scale, prospective and randomized studies are required for further confirmation of these results.

## 5 CONCLUSIONS

In summary, receiving standard R‐CHOP treatment without prednisone combined with antacids and anti‐Hp therapy before a gastric ulcer is cured could be an effective regimen for localized PG‐DLBCL. Our study indicates that decreasing the risk of hemorrhage and perforation during immunochemotherapy improves the EFS and OS of patients with localized PG‐DLBCL but not of patients with disseminated disease. Therefore, preventative treatment could be applied in the treatment of gastric cancer and other subtypes of gastric lymphoma. Further prospective and large‐scale research is needed to confirm these findings.

## AUTHOR CONTRIBUTIONS

**Limei Zhang:** Formal analysis (lead); writing – original draft (lead); writing – review and editing (equal). **He Huang:** Methodology (equal); project administration (equal); software (equal); supervision (equal). **Zhao Wang:** Data curation (equal); visualization (equal); writing – review and editing (equal). **Xiaojie Fang:** Data curation (equal); methodology (equal); software (equal); supervision (equal). **Huangming Hong:** Investigation (equal); methodology (equal); writing – review and editing (equal). **Yongchang Chen:** Supervision (supporting); visualization (supporting). **Quanguang Ren:** Resources (supporting). **Yuyi Yao:** Resources (supporting); supervision (supporting). **Zegeng Chen:** Resources (supporting); supervision (supporting). **Fei Pan:** Resources (supporting); supervision (supporting). **Xiaoqian Li:** Supervision (supporting). **Meiting Chen:** Methodology (supporting); visualization (supporting). **Tong‐Yu Lin:** Conceptualization (equal); funding acquisition (lead); project administration (lead); resources (lead); writing – review and editing (lead).

## CONFLICT OF INTEREST

No conflicts of interest.

## ETHICAL APPROVAL

This study was approved by the ethics committee of Sun Yat‐sen University Cancer Center. Our study abided by the principles of the Declaration of Helsinki. The requirement for informed consent from patients was waived because this was a retrospective study.

## Data Availability

Data sharing is not available because of the lack of new data in this study.
